# In Obesity, HPA Axis Activity Does Not Increase with BMI, but Declines with Aging: A Meta-Analysis of Clinical Studies

**DOI:** 10.1371/journal.pone.0166842

**Published:** 2016-11-21

**Authors:** Judit Tenk, Péter Mátrai, Péter Hegyi, Ildikó Rostás, András Garami, Imre Szabó, Margit Solymár, Erika Pétervári, József Czimmer, Katalin Márta, Alexandra Mikó, Nóra Füredi, Andrea Párniczky, Csaba Zsiborás, Márta Balaskó

**Affiliations:** 1 Institute for Translational Medicine, Medical School, University of Pécs, Pécs, Hungary; 2 Institute of Bioanalysis, Medical School, University of Pécs, Pécs, Hungary; 3 Department of Translational Medicine, University of Pécs, Pécs, Hungary; 4 Hungarian Academy of Sciences - University of Szeged, Momentum Gastroenterology Multidisciplinary Research Group, Szeged, Hungary; 5 Department of Gastroenterology, First Department of Internal Medicine, University of Pécs, Pécs, Hungary; Charité-Universitätsmedizin Berlin, Campus Benjamin Franklin, GERMANY

## Abstract

**Background:**

Obesity is one of the major public health challenges worldwide. It involves numerous endocrine disorders as etiological factors or as complications. Previous studies strongly suggested the involvement of the hypothalamic-pituitary-adrenal (HPA) axis activity in obesity, however, to date, no consistent trend in obesity-associated alterations of the HPA axis has been identified. Aging has been demonstrated to aggravate obesity and to induce abnormalities of the HPA axis. Thus, the question arises whether obesity is correlated with peripheral indicators of HPA function in adult populations.

**Objectives:**

We aimed to meta-analyze literature data on peripheral cortisol levels as indicators of HPA activity in obesity during aging, in order to identify possible explanations for previous contradictory findings and to suggest new approaches for future clinical studies.

**Data Sources:**

3,596 records were identified through searching of PubMed, Embase and Cochrane Library Database. Altogether 26 articles were suitable for analyses.

**Study Eligibility Criteria:**

Empirical research papers were eligible provided that they reported data of healthy adult individuals, included body mass index (BMI) and measured at least one relevant peripheral cortisol parameter (i.e., either morning blood cortisol or 24-h urinary free cortisol).

**Statistical Methods:**

We used random effect models in each of the meta-analyses calculating with the DerSimonian and Laird weighting methods. I-squared indicator and Q test were performed to assess heterogeneity. Meta-regression was applied to explore the effect of BMI and age on morning blood and urinary free cortisol levels. To assess publication bias Egger’s test was used.

**Results:**

Obesity did not show any correlation with the studied peripheral cortisol values. On the other hand, peripheral cortisol levels declined with aging within the obese, but not in the non-obese groups.

**Conclusions:**

Our analysis demonstrated that obesity or healthy aging does not lead to enhanced HPA axis activity, peripheral cortisol levels rather decline with aging.

## Introduction

Obesity, one of the major public health challenges of the world involves a great variety of endocrine disorders as etiological factors or as complications [1]. Previous studies suggested a potential involvement of the hypothalamic-pituitary-adrenal (HPA) axis in obesity [2, 3]. Regarding the HPA axis, peripheral glucocorticoid hyperfunction is thought to be a major factor in correlation with obesity, either as an etiological factor of adiposity [3] or as a complication of weight gain [2]. On the other hand, central members of the axis would rather induce weight loss [4–7]. In particular, hypothalamic corticotropin-releasing factor (CRF) the major activator of the HPA axis is a well-known anorexigenic mediator [4, 5] and pro-opiomelanocortin, the precursor of adrenocorticotropin hormone (ACTH released from the anterior pituitary upon CRF activation) also has anorexigenic derivatives, such as alpha-melanocyte stimulating hormone [6, 7]. The potential involvement of glucocorticoids in obesity is supported by numerous factors (for review see [2, 3]). Among other actions, they induce gluconeogenesis leading to hyperglycemia and hyperinsulinemia promoting fat deposition in case of enhanced caloric intake, they promote the differentiation and proliferation of human adipocytes [8], and they also play a role in the development of metabolic syndrome associated with visceral obesity [9]. Such findings raise the possibility that visceral fat accumulation and enhanced activity of the HPA axis may be linked to each other, but characteristics of this relationship remain unknown [10].

Previous findings do not show consistent trends in obesity-related to HPA axis alterations. While both positive and negative correlations have been found by different clinical observations, the most consistent feature reported by a number of studies was a negative linear relationship between body mass index (BMI) and morning serum or salivary cortisol levels [11–16]. Interestingly, one study described even a non-linear, U-shaped relationship between BMI and glucocorticoids: circadian slopes of salivary cortisol secretion were lowest in mildly overweight individuals (nadir), whereas high values were observed both in the severely obese and in the low-normal BMI groups [14].

Aging is a major factor that influences HPA axis activity [17, 18] and at the same time, it is also known to increase the prevalence of obesity reaching a peak in middle-aged populations [19]. No correlation of aging or BMI versus cortisol levels were described in healthy lean individuals [20, 21, 22]. In obesity, however a gradual increase in integrated daily cortisol release has been described during the course of aging [23].

We aimed to review the literature on peripheral cortisol levels as indicators of HPA activity in healthy adult individuals (above 18 years) with or without obesity during aging, complemented by a meta-analysis of the available human data, in order to identify possible explanations for previous contradictory findings and to suggest possible new approaches for future clinical studies.

## Methods

### Search strategy and study selection

**Fig 1:**
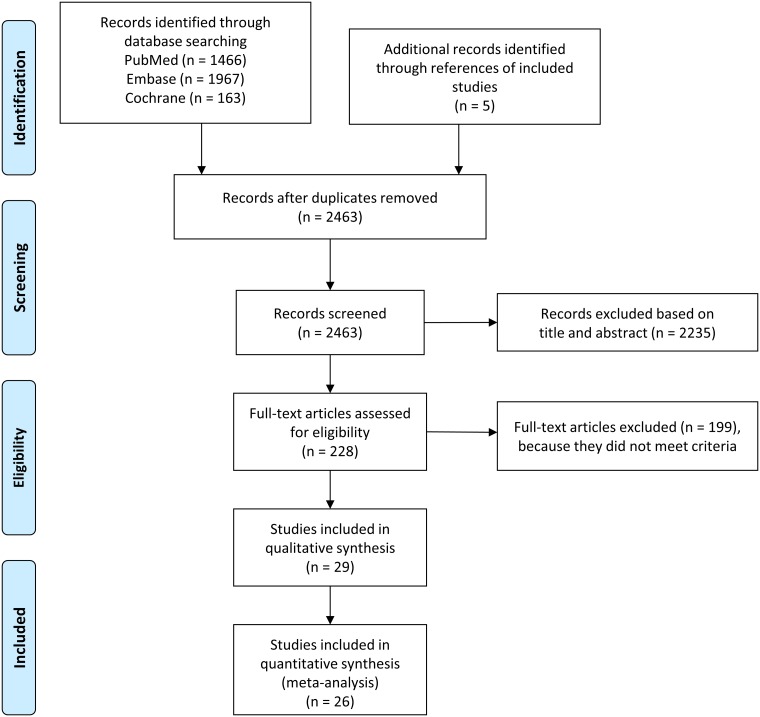
Flowchart of the study selection procedure.

We performed this study following principles of the PRISMA statement [24]. No review protocol was registered for this meta-analysis. Database search was conducted in May 2016. Our meta-analysis was based on the PICO format (P: adult individuals older than 18 years, I: mild/severe obesity, C: normal body weight O: morning serum/plasma cortisol, 24- h urinary cortisol). Records (altogether 3596) were identified through searching of PubMed (http://www.ncbi.nlm.nih.gov/pubmed), Embase (https://www.embase.com) and Cochrane Library Database (http://www.cochranelibrary.com) (Fig 1). All together twenty-six articles were suitable for analyses [25–50]. The following search strings were used alone and in combination: ‘obesity’ and ‘obese’ and ‘overweight’ and ‘serum cortisol’ and ‘plasma cortisol’and ‘morning cortisol and ‘urinary cortisol’ and ‘UFC’. Search strategy of PubMed was as follows: (‘obesity’ OR ‘obese’ OR ‘overweight’) AND ‘(morning cortisol OR serum cortisol OR plasma cortisol)’; (‘obesity’ OR ‘obese’ OR ‘overweight’) AND (‘urinary cortisol’ OR ‘UFC’). This search identified 1466 records.

Exclusion criteria included animal experiments, non-English language reports, steroid treatment, endocrine diseases affecting the HPA axis (such as Cushing’s disease or syndrome, androgen hyperactivity, polycystic ovary syndrome), pregnancy and psychiatric disorders affecting the HPA axis (such as depression, anxiety, anorexia nervosa, etc.).

Clinical studies were eligible provided that they reported data of adult, predominantly healthy individuals and included BMI and measured at least one relevant peripheral cortisol parameter (i.e. either morning blood cortisol or 24-h urinary free cortisol).

Regarding assessment of risk of bias of individual studies, the fact that very few such reports were available in the literature, precluded exclusion of them based on lack of randomized design or low participant numbers. Such a risk of bias was relatively low, in any case. Our study focused on correlations between physiological parameters, such as periheral cortisol levels and BMI and/or age not on treatment efficacy, therefore blinding (to exclude placebo effects) was of relatively minor relevance. Moreover, due to weighting methods, data with low participant numbers were assigned with lower weights during the analysis.

### Data extraction and evaluation procedure

Two authors (J.T., I.R.) independently performed study selection by following inclusion criteria as indicated above. The following pieces of information were recorded for each study: first author, publication year, sample size, gender, age, BMI, mean values of morning blood cortisol in μg/dL and/or 24h- urinary free cortisol in μg/dL (Table 1). No supplementary information was obtained from investigators of the original clinical studies, only published data were used.

**Table 1:** Description of the studies included in the meta-analyses.

Study	Sample size	Gender	Age	Weight group *	Morning blood cortisol (μg/dL)	24-h urinary free cortisol (μg/dL)	Relationship between BMI and cortisol	
			Mean		Mean ± SD	Mean ± SD	Morning blood cortisol	24-h urinary free cortisol
**Arikan et al. (2009)** [25]	30	female	24,64	N	12.28 ± 5.09	NR	NR	NR
**Barat et al. (2005)** [26]	44	female	33,00	SO	17.51 ± 5.11	NR	NR	NR
**Bentley-Lewis et al. (2007)** [27]	57	both	43,00	MO	12.10 ± 3.77	58.50 ± 30.51	not significant	not significant
	63		37,00	N	12.50 ± 3.97	63.90 ± 50.58		
**Brumby et al. (2013)** [28]	37	female	53,22	MO	13.55 ± 3.97	NR	NR	NR
**Casulari et al. (2015)** [29]	16	both	34,60	MO	7.19 ± 3.35	NR	NR	NR
**Cizza et al. (2012)** [30]	44	both	35,30	N	19.52 ± 8.50	62.19 ± 22.58	NR	NR
**Constantinopoulos et al. (2015)** [31]	18	both	34,70	SO	10.7 ± 4.10	71.30 ± 62.70	NR	NR
**Drent et al. (1995)** [32]	24	both	46,70	MO	13.24 ± 4.32	NR	NR	NR
**Duclos et al. (2014)** [33]	22	female	33,63	SO	17.46 ± 5.05**	38.68 ± 16.29**	NR	not significant
	31		35,40					
**Engeli et al. (2004)** [34]	23	female	56,00	N	12.70 ± 6.10	19.00 ± 9.00	NR	NR
	23		57,00	MO	10.50 ± 40	24.00 ± 10.00		
	24		58,00	SO	8.90 ± 3.30	22.00 ± 11.00		
**Gambineri et al. (2014)** [35]	20	female	28,70	MO	12.43 ± 4.70	142 ± 58.14	NR	NR
**Hallschmid et al. (2006)** [36]	10	both	NR (range: 23–46)	MO	7.77 ± 2.53	NR	NR	NR
	12			N	5.88 ± 2.08			
**Prpić-Križevac et al. (2012)** [37]	29	both	49,40	MO	15.78 ± 4.92	57.79 ± 27.14	not significant	not significant
	19		46,10	N	17.96 ± 6.2	55.04 ± 21.34		
**Labouesse et al. (2014)** [38]	25	female	31,70	MO	11.60 ± 4.6	NR	NR	NR
**Mota Martins et al. (2014)** [39]	40	female	28,15	N	9.89 ± 5.61	NR	NR	NR
	40		32,33	SO	9.56 ± 4.86			
**Monteleone et al. (2003)** [40]	31	female	23,80	N	12.30 ± 4.80	NR	NR	NR
	12		37,40	SO	120 ± 3.10			
**Müssig et al. (2008)** [41]	72	both	39,30	SO	11.75 ± 5.94	39.90 ± 23.76	NR	NR
	30		30,30	N	NR	40.10 ± 19.72		
**Olszanecka-Glinianowicz et al. (2007)** [42]	34	female	31,00	SO	13.66 ± 9.51	NR	NR	NR
**Pasquali et al. (2002)** [43]	13	male	35,60	N	13.33 ± 4.39**	NR	NR	NR
	21	female	31,60					
	21	male	39,70	SO	13.00 ± 5.09**			
	51	female	34,70					
**Putignano et al. (2001)** [44]	47	female	35,50	MO	13.90 ± 4.94	43.51 ± 27.08	negative linear relationship p<0.05	not significant
	103		38,20	SO	12.20 ± 5.78	37.72 ± 21.01		
	63		31,20	N	15.20 ± 8.57	47.10 ± 29.37		
**Rask et al. (2001)** [45]	11	male	46,80	N	10.33 ± 2.86	NR	NR	NR
	11		49,60	MO	11.54 ± 3.87**			
	12		51,90					
**Schorr et al. (2015)** [46]	21	female	30,00	MO	18.30 ± 6.80	67.00 ± 35.20	U-shaped relationship R = 0.45 p = 0.003	U-shaped relationship R = 0.47 p = 0.004
	21		27,00	N	21.90 ± 8.90	51.10 ± 18.80		
**Shabir et al. (2013)** [47]	55	female	25,00	N	11.30 ± 4.50	NR	NR	NR
**Stewart et al. (1999)** [48]	12	both	33,10	MO	16.35 ± 2.24**	47.75 ± 5.31 **	not significant	not significant
	12		33,90					
	12		27,90	N	16.93 ± 1.30	47.10 ± 7.50		
**Tomiyama et al. (2014)** [49]	47	female	40,89	MO	9.97 ± 1.48	NR	NR	NR
**Vicennati et al. (2009)** [50]	127	female	44,80	SO	NR	38.20 ± 23.60	NR	positive linear relationship p<0.001
	21		34,00	N		21.10 ± 9.80		

BMI, body mass index; NR, not reported

*Weight group indicates non-obese = N (BMI < 25), mildly obese = MO (25 < BMI < 35) or severely obese = SO (BMI > 35) participant groups

** indicate merged data

### Statistical methods

We used random effect models in each of the meta-analyses calculating with the DerSimonian and Laird weighting method. Regarding summary measures (PRISMA [24]), primary analyses demonstrate differences of means with 95% confidence intervals (95% CI) from studies that contained data for obese and non-obese control groups, as well. Addititional analyses show mean cortisol values of subgroups with 95% CI intervals (with summarized weighted means). These groups of the clinical studies were assigned to non-obese (N, BMI < 25), mildly obese (MO, 25 < BMI < 35) or severely obese (SO, BMI > 35) categories.

To assess whether the heterogeneity observed among effect sizes could be attributed to random chance or other factors (different clinical methods or diverse participants), I-squared indicator and Q test were performed. I-squared statistics represents the percentage of effect size heterogeneity that cannot be explained by random chance but that is determined by other factors (mentioned above). If the Q test is significant, it implies that the heterogeneity among effect sizes reported in the observed studies are more diverse than it could be explained only by random error. We considered the Q test significant if p < 0.1.

We used meta-regression models to explore the effect of various factors on morning and urinary free cortisol levels. In each case we tested the whole model (simultaneously hypothesized that all coefficients are zero) and report the regression coefficients, 95% CI-s, standard errors and z tests. We also calculated the explained variance of the model (R^2^ analog) and the result of the Q test to evaluate if the unexplained variance is zero.

To compare the cortisol levels of non-obese, mildly and severely obese groups, we used subgroup analysis, p < 0.05 indicating significant difference.

To assess the presence of publication bias, we used Egger’s test to detect asymmetry in the funnel plot. A significant test result (p < 0.1) indicates the existence of bias.

Some studies reported the data (age, cortisol, etc.) separately for men and women or for different BMI groups. In these cases, it was possible to calculate the variance and the mean of the whole group using the additivity of within-group sum of squares and between-group sum of squares. From the sum of squares, variance was calculated. We report every instance where such calculation was conducted (Table 1).

All statistical analyses were performed with Comprehensive Metaanalysis Software (Biostat Inc.) and Stata 11 SE (Stata Corp.).

## Results

### Search results

In May 2016 3596 records were identified through database searching based on PICO (P: adult individuals older than 18 years, I: mild/severe obesity, C: normal body weight O: morning serum/plasma cortisol, 24- h urinary cortisol) from PubMed (n = 1466), from Embase (n = 1967) and from Cochrane Library database (n = 167) and screened for duplicates (Fig 1). From other sources (from reference lists of screened studies) 5 additional records were obtained. Following further screening of non-English language articles, animal background, lack of abstract or full-text availability and exclusion based on title and abstract evaluation, 228 records remained for detailed assessment of eligibility. Altogether 29 articles were chosen [25–50], but finally only 26 proved to be eligible for statistical analysis, based on inadequate data presentation (Fig 1).

### Study characteristics

Studies used in our meta-analysis dated from 1995 to 2015. Data of 1511 individuals were included in our analysis (number of participants ranged from 16 to 213 per study). From each study sample size, gender, age, BMI, mean values of morning blood cortisol and/or 24h- urinary free cortisol were extracted (Table 1).

Relatively few studies reported such values. Therefore we decided to include all data in our meta-analysis without exclusion based on assessment of risk of bias of individual studies. This risk of bias appeared to be low, since our study focused on correlations between physiological parameters (periheral cortisol levels versus BMI and/or age) and not on treatment efficacy. In our case, blinding of participants, to exclude placebo effects, was of minor importance. Due to weighting methods, data with low participant numbers were assigned with lower weights in our analysis.

Among the twenty-six studies only seven [27, 33, 37, 44, 46, 48, 50] investigated the relationship between BMI and HPA axis activity based on 24-h urinary free cortisol. In addition five studies of the seven also measured morning blood cortisol [27, 37, 44, 46, 48]. In others no correlation between these values was studied. Only one study found significant linear correlation between obesity and morning blood cortisol of female participants [44], and one work focusing also on female volunteers reported non-linear U-shaped relationship between morning blood cortisol or 24-h urinary free cortisol and BMI [46]. All other studies failed to reveal any association between these values.

Regarding gender, the majority of the studies (fifteen of them) contained data regarding morning blood cortisol and/or 24-h urinary free cortisol of women [25, 26, 28, 33–35, 37–39, 41, 44, 46, 47, 49, 50], four of which also tested the correlation between BMI and cortisol values [33, 44, 46, 50]. Two of them found association between obesity and peripheral cortisol [44, 46]. One study reported morning blood cortisol data of male participants without analysing potential correlation [45], and the remaining ten clinical studies pooled data of male and female volunteers [27, 29–32, 36, 40, 42, 43, 48].

Regarding age, in most studies, mean age value (with SD) was given for a wide age-range of the participants. This mean age exceeded 50 years only in three of the studies [28, 34, 45]. None examined association between cortisol values and age.

### Primary analysis and publication bias

**Fig 2:**
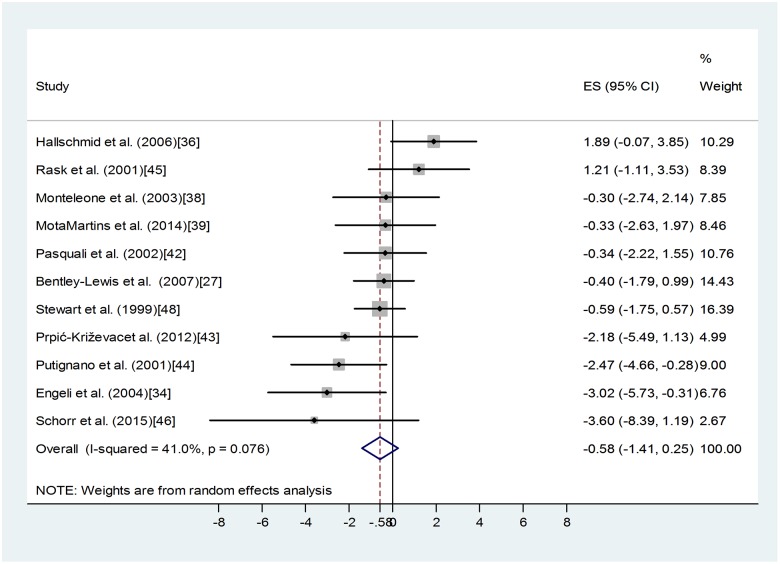
Forest plot representing the differences in mean morning blood cortisol values of obese and non-obese groups. Squares show the difference in mean values with the grey area reflecting the weight assigned to the study. Horizontal bars indicate 95% confidence intervals (95% CI). The diamond shows the overall effect size (ES) with its corresponding 95% CI.

Regarding those eleven studies that contained values of morning blood cortisol of obese and non-obese groups [27, 34, 36, 38, 39, 42–46, 48] the classic random effect model showed no effect of obesity on this parameter in otherwise healthy adults (Fig 2). Weighted overall effect size (ES) = -0.58 with 95% confidence interval (95% CI of -1.41, 0.25), p = 0.173. Heterogeneity of the data was high: I-squared = 40.97%, p = 0.076, indicating the presence of other determining factors in the background.

**Fig 3:**
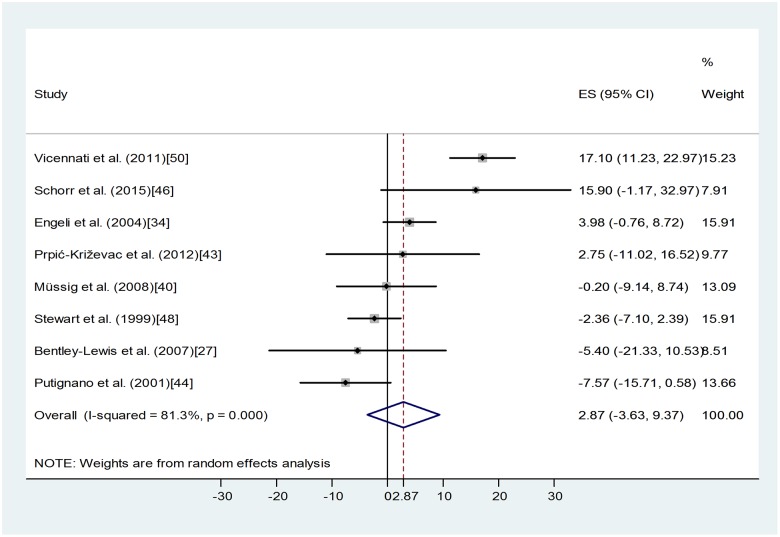
Forest plot representing the differences in mean 24-h urinary free cortisol values of obese and non-obese groups. Squares show the difference in mean values with the grey area reflecting the weight assigned to the study. Horizontal bars indicate 95% confidence intervals (95% CI). The diamond shows the overall effect size (ES) with its corresponding 95% CI.

Similar meta-analysis concerning 24-h urinary free cortisol (eight studies, [27, 34, 40, 43, 44, 46, 48, 50]) demonstrated a similar lack of overall effect (Fig 3). ES = 2.87 95% CI (-3.63, 9.37), p = 0.387. Heterogeneity of the data was high: I-squared = 81.3%, p < 0.0001, even more strongly indicating the presence of other determining factors in the background.

No publication bias was identified using Egger’s test: p = 0.262 for morning blood cortisol and p = 0.266 for 24-h urinary free cortisol.

### Additional analyses

Weighted mean peripheral cortisol values of N, MO or SO participant groups of all studies (with or without calculation of correlation between BMI and peripheral cortisol) were also analysed by subgroup analysis.

**Fig 4:**
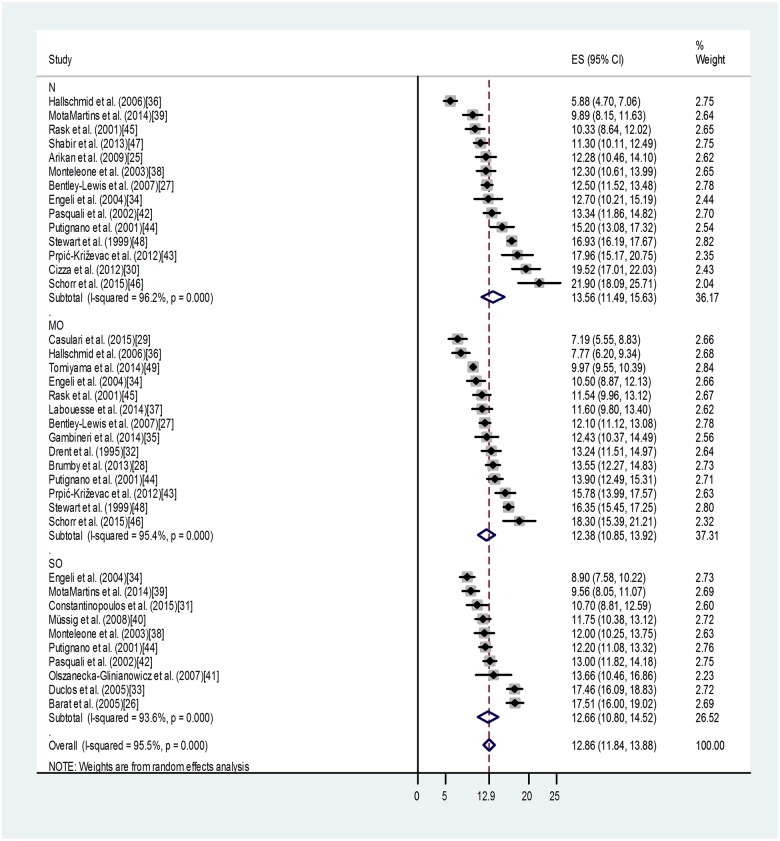
Forest plot indicating the mean values of morning blood cortisol in non-obese (N), mildly (MO) or severely obese (SO) groups. The grey areas reflect the weight assigned to the study. Horizontal bars indicate 95% confidence interval (95% CI). The diamond indicates the weighted mean of each subgroup with the corresponding 95% CI. ES: effect size.

As shown in Fig 4, we found a lack of effect of mild or severe obesity on morning blood cortisol. For category N: overall weighted mean was 13.56 with 95% CI (11.49, 15.63), relative weight 36.17%. For category MO: overall weighted mean was 12.38 with 95% CI (10.85, 13.92), relative weight 37.31%. For category SO: overall weighted mean was 12.66 with 95% CI (10.80, 14.52), relative weight 26.52%. High value of heterogeneity (I-squared: 95.50%, p < 0.0001) for morning blood cortisol, indicates the contribution of other strong factors determining these values.

**Fig 5:**
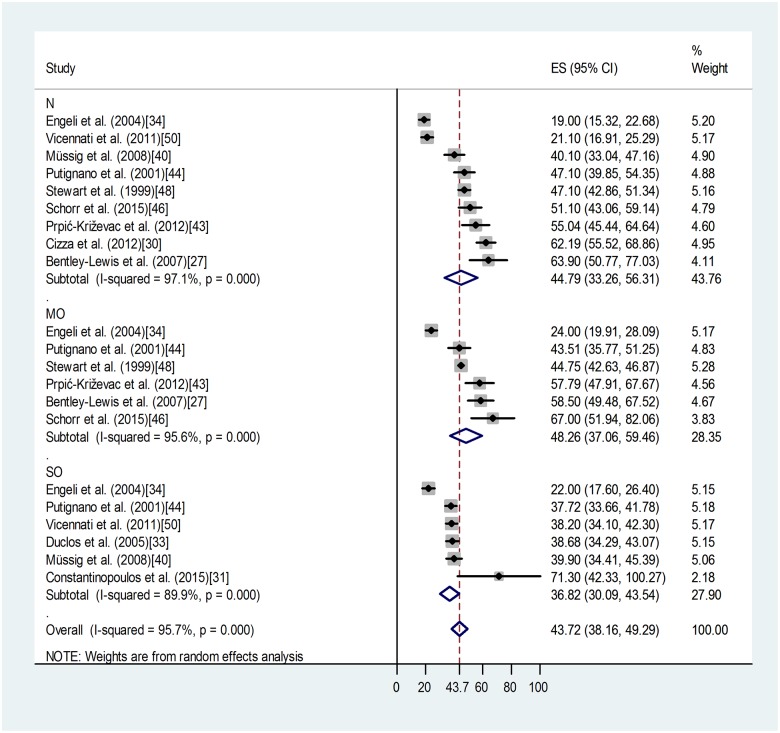
Forest plot indicating the mean values of 24-h urinary free cortisol in non-obese (N), mildly (MO) or severely obese (SO) groups. The grey areas reflect the weight assigned to the study. Horizontal bars indicate 95% confidence interval (95% CI). The diamond indicates the weighted mean of each subgroup with the corresponding 95% CI. ES: effect size.

Fig 5 demonstrates a lack of effect of mild or severe obesity on 24-h urinary cortisol. For category N: overall weighted mean was 44.79 with 95% CI (33.26, 56.31), relative weight 43.76%. For category MO: overall weighted mean was 48.26 with 95% CI (37.06, 59.46), relative weight 28.35%. For category SO: overall weighted mean was 36.82 with 95% CI (30.09, 43.54), relative weight 27.90%. High value of heterogeneity (I-squared: 95.70%, p < 0.0001) for 24-h urinary free cortisol indicates the contribution of other strong factors determining these values.

Meta-regressions regarding correlation between BMI and morning blood cortisol or 24-h urinary cortisol values also failed to show any singnificant effect (results not shown).

**Fig 6:**
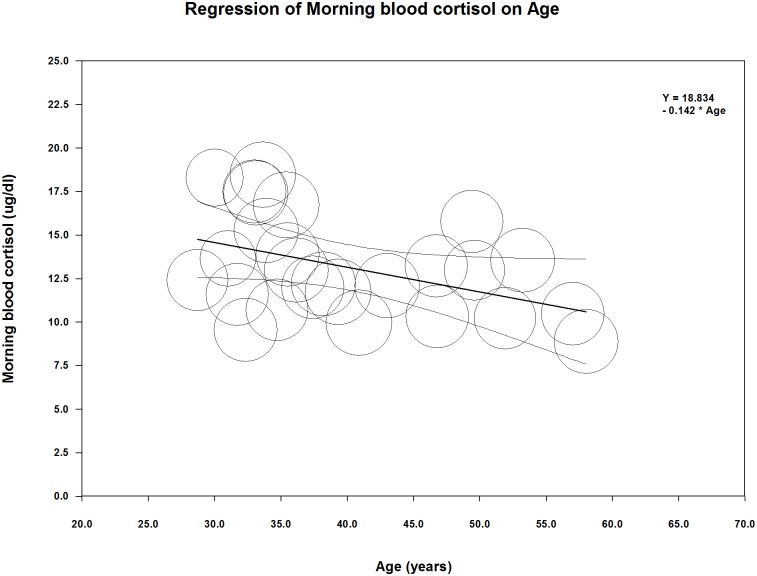
Meta-regression of mean morning blood cortisol levels versus mean age of the 26 obese groups from our analyzed studies (Table 1).

Concerning the effects of aging, meta-regression showed a slight but significant decline of morning blood cortisol between 30 to 60 years of age (Fig 6). This negative correlation was observed within the obese (number of groups: 26, coefficient: -0.14, p = 0.023, r-square analogue: 0.34) and also in the merged groups (number of groups: 40, coefficient: -0.11, p = 0.027, r-square analogue: 0.25), but not within the non-obese population (number of groups: 14, coefficient: -0.02, p = 0.860). However, in both cases of significant linear correlations, the goodness of fit test indicates that the unexplained variance is not zero (for the obese groups: Q = 1110.19, df = 24, p < 0.0001, for the merged groups: Q = 1547.27, df = 38 p < 0.0001), suggesting other determinig factors in the background.

A similar relationship (not shown) was observed between 24-h urinary free cortisol and aging within the obese (number of groups: 15, coefficient: -0.78, p = 0.002, r-square analogue: 0.4) and the merged groups (number of groups: 24, coefficient: -0.93 p = 0.0007, r-square analogue: 0.28), but not among normal, non-obese participants (number of groups: 9, coefficient: -0.61, p = 0.315). However, in both cases of significant linear correlations, the goodness of fit test indicates that the unexplained variance is not zero (for the obese groups: Q = 120.31, df = 13, p < 0.0001, for the merged groups: Q = 312.5, df = 22, p < 0.0001), suggesting the role of other determinig factors.

## Discussion

Our present study aimed to review the literature on markers of peripheral cortisol release (morning blood cortisol and 24-h urinary free cortisol) as indicators of HPA axis activity in obesity during aging in otherwise healthy, non-depressed adults, complemented by a meta-analysis of the available human data. We intended to identify possible explanations for previous contradictory findings and to suggest new methodological approaches for future clinical studies.

Our results show that in predominantly healthy non-obese adults, BMI fails to influence morning blood cortisol or 24-h urinary free cortisol values. Although experimental data continues to suggest that excess adiposity and obesity result in increased adrenal cortisol secretion [2], meta-regression focusing on correlation between pooled BMI values (of non-obese, mildly or severely obese populations) and peripheral (morning blood, or 24-h urinary) cortisol values, failed to show significant effects. No such correlation was revealed in any of the obese subgroups either. These results are in contrast with findings of previous studies showing either decreased cortisol levels in obesity [11, 13–16, 51] or non-linear U-shaped correlation of peripheral cortisol and BMI accross the BMI spectrum [46]. Moreover, high heterogeneity of our data indicates that excessive variations in the data are not accidental, but suggests the presence of other determining factors in the background. Such determining factors could include general life stress of the participants, unhealthy diet, light conditions or impaired sleep quality (the latter two especially affects morning blood cortisol) [3, 52, 53], etc. Gender could also be an important confounder [3, 54, 55]. In most available studies blood and urine samples were collected from males and females and results were pooled. Gender differences could therefore contribute to inconsistent trends in the results. In females, follicular or luteal phase of the menstrual cycle, as well as pre- or post-menopausal status are also known to influence the HPA axis activity [33, 56, 57]. Importance of such additional determining factors that may influence peripheral cortisol release, independently of visceral obesity, are emphasized by previous clinical findings [9, 31]. These previous studies report that higher cortisol levels appear to induce metabolic syndrome in people suffering of severe visceral obesity, whereas similarly obese individuals with low cortisol output do not show characteristics of metabolic syndrome [9, 31]. Thus, high cortisol output appears to aggravate complications of obesity.

Regarding aging, our analysis showed an age-related decline in peripheral cortisol values from 30 to 60 years of age within the obese group. No such correlation was found in healthy individuals with normal BMI. Our results are in accord with previous observations based on a relatively young cohort (mean age 33 ± 9.8) also showing age-related decline in cortisol values [58]. These results, however, appear to contradict other reports that demonstrated increased or maintained activity of the HPA axis during aging presumably participating in the development of depression and frailty in the elderly [59–62]. Other investigators also suggested a role for the hyperactive HPA axis in neuronal deterioration of aged humans and in old experimental animals [59, 63]. Our results are also contradictory with those works that reported a gradual increase in integrated cortisol during the course of aging in obesity [23]. In conclusion, our analysis suggests that aging *per se* does not necessarily lead to increased HPA axis activity in healthy populations with or without obesity, but it will rather be associated with some decline of peripheral cortisol output. Such a reduction in HPA axis activity during otherwise healthy aging in obese populations, would slow down further progression of obesity and that of related co-morbidities (such as type 2 diabetes mellitus or hypertension, etc.). On the other hand, increases in HPA axis activity in obese or aging populations due to stress, sleep disorders, unhealthy diet or other, as yet, undefined factors would promote the progression of obesity and related co-morbidities [3, 52, 53]. Thus, our findings underline the importance of such factors, the role of which may be more important in the development of obesity- or age-related complications than that of obesity- or age-associated dysregulation of the HPA axis [46, 64].

It also has to be emphasized that our meta-analysis has numerous limitations mainly because of the restricted availability of publications reporting suitable data. In our meta-analysis, only BMI was available for the assessment of obesity, since very few studies reported appropriate data on fat content and fat distribution. Among them, visceral fat ratio could have been the value most likely affected by variations in the HPA axis activity [53, 65]. Unfortunately, we could not use such values in our analysis, due to insufficient data availability. Future studies should be aimed to determine the correlation between visceral fat ratio and HPA axis activity during the course of aging. In addition, among the twenty-six studies that we analyzed only seven focused on the relationship between BMI and HPA axis activity based on 24-h urinary free cortisol [27, 33, 37, 44, 46, 48, 50]. Five of these seven studies also reported morning blood cortisol [27, 37, 44, 46, 48]. In the other nineteen studies no correlation between these values was tested. Regarding the assessment of HPA axis activity, initially we have considered a number of potential variables also including cortisol awakening response curves (CAR, based on salivary cortisol) [66], diurnal cortisol slope [67] and also hair cortisol (reflecting long-term cortisol output) [68] as indicators. Once again, insufficient availability of data limited our choice to morning blood cortisol (that is a technically simpler but less sensitive tool to evaluate HPA axis activity than CAR) and 24-h urinary free cortisol (that reflects overall daily cortisol output, albeit with high variations). Standardized evaluation of HPA axis activity in future studies would greatly enhance the reliability and value of future meta-analyses. Regarding gender, the majority of the studies (fifteen of them) contained data regarding morning blood cortisol or 24-h urinary free cortisol of women [25, 26, 28, 33–35, 37–39, 41, 44, 46, 47, 49, 50], four of which also tested the correlation between BMI and cortisol [33, 44, 46, 50]. Two of them found some association between obesity and morning blood cortisol or urinary free cortisol [44, 46]. One study reported morning blood cortisol data of male participants without analyzing potential correlation [45], and the remaining ten clinical observations pooled data of male and female volunteers [27, 29–32, 36, 40, 42, 43, 48]. These statistics suggest that an association between peripheral cortisol output and BMI would be more probably found in studies focusing on women.

Concerning aging, hardly any paper was found that focused on old populations. Mean age of the participants exceeded 50 years only in three of the studies [28, 34, 45]. None examined association between cortisol values and BMI. In the available studies a high heterogeneity of age was found that could also contribute to some of the inconsistencies. In most studies, only mean age value (with SD) was given for a wide age-range of the participants limiting the strength of any correlation involving age.

Genetic, obesity- or age-related alterations in peripheral cortisol metabolism in muscles or in adipose tissue may also contribute to confounders [34, 65, 69, 70]. Such alterations may involve changes in the activity of 11-beta-hydroxysteroid dehydrogenase type I (activation) or type II (deactivation). Some studies described an enhancement of 11-beta-hydroxysteroid dehydrogenase type I (activation) activity in obesity [34] and also during the course of aging [67, 71]. However, no conclusive evidence clarifies the role of these enzymes in obesity or aging.

Concerning limitations of previous studies and potential explanation of the diverse findings, we suggest that different sample techniques and assay methods may contribute to high variations, especially in 24-h urinary free cortisol values. Moreover, 24-h urinary free cortisol measurements were not corrected for creatinine excretion or body surface area. Body composition (fat mass) data were not included either in the available studies, which could also contribute to the limited strength of the results. Small number of participants also limited the weight of the results in most studies.

In conclusion, findings of our meta-analysis suggest that neither obesity, nor healthy aging is necessarily associated with increased HPA axis activity (as indicated by peripheral cortisol output) in otherwise healthy populations. Peripheral cortisol levels rather decline with aging. However, since increased peripheral cortisol output aggravates consequences of both obesity and aging, investigation of other determinig factors of HPA axis activity is of outstanding importance.

## Supporting Information

S1 File PRISMA checklist of the meta-analysis. (PDF)
